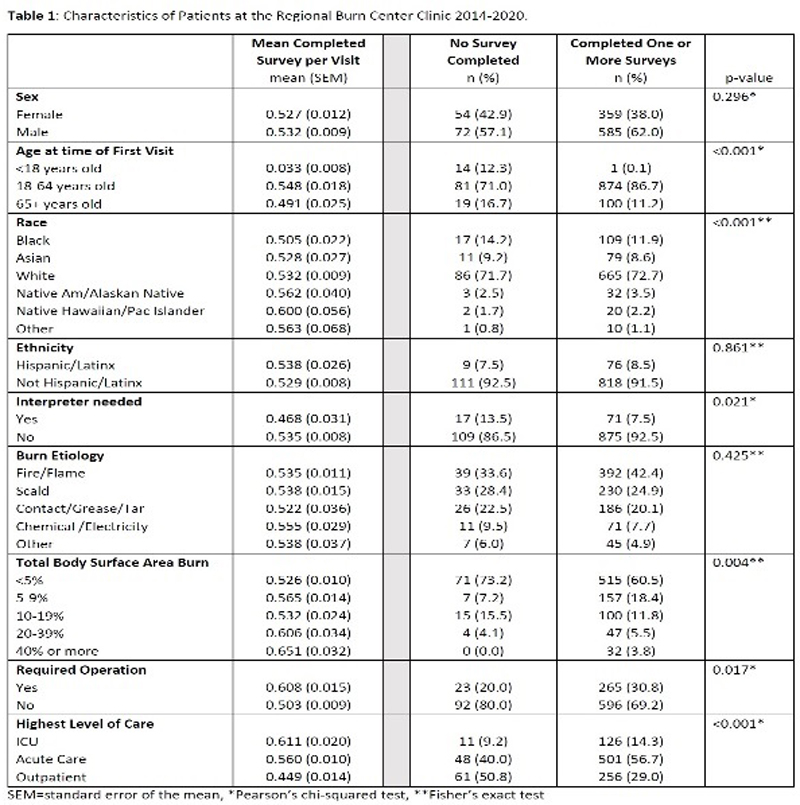# 731 Evaluation of PROMIS-10 Global Health Survey Implementation at a Regional Burn Center Clinic

**DOI:** 10.1093/jbcr/irad045.206

**Published:** 2023-05-15

**Authors:** Mary Hunter, Devin Gaskins, Michelle Hubbard, Gretchen Carrougher, Shelley Wiechman, Barclay Stewart, Mona Chambers, Nicole Gibran

**Affiliations:** UW Medicine Regional Burn Center, Seattle, Washington; University of Washington School of Medicine, Seattle, Washington; UW Medicine Regional Burn Center, Seattle, Washington; University of Washington, Seattle, Washington; Department of Rehabilitation Medicine, University of Washington School of Medicine, Seattle, Washington; University of Washington, Seattle, Washington; UW Medicine Regional Burn Center, Seattle, Washington; University of Washington, Seattle, Washington; UW Medicine Regional Burn Center, Seattle, Washington; University of Washington School of Medicine, Seattle, Washington; UW Medicine Regional Burn Center, Seattle, Washington; University of Washington, Seattle, Washington; Department of Rehabilitation Medicine, University of Washington School of Medicine, Seattle, Washington; University of Washington, Seattle, Washington; UW Medicine Regional Burn Center, Seattle, Washington; University of Washington, Seattle, Washington; UW Medicine Regional Burn Center, Seattle, Washington; University of Washington School of Medicine, Seattle, Washington; UW Medicine Regional Burn Center, Seattle, Washington; University of Washington, Seattle, Washington; Department of Rehabilitation Medicine, University of Washington School of Medicine, Seattle, Washington; University of Washington, Seattle, Washington; UW Medicine Regional Burn Center, Seattle, Washington; University of Washington, Seattle, Washington; UW Medicine Regional Burn Center, Seattle, Washington; University of Washington School of Medicine, Seattle, Washington; UW Medicine Regional Burn Center, Seattle, Washington; University of Washington, Seattle, Washington; Department of Rehabilitation Medicine, University of Washington School of Medicine, Seattle, Washington; University of Washington, Seattle, Washington; UW Medicine Regional Burn Center, Seattle, Washington; University of Washington, Seattle, Washington; UW Medicine Regional Burn Center, Seattle, Washington; University of Washington School of Medicine, Seattle, Washington; UW Medicine Regional Burn Center, Seattle, Washington; University of Washington, Seattle, Washington; Department of Rehabilitation Medicine, University of Washington School of Medicine, Seattle, Washington; University of Washington, Seattle, Washington; UW Medicine Regional Burn Center, Seattle, Washington; University of Washington, Seattle, Washington; UW Medicine Regional Burn Center, Seattle, Washington; University of Washington School of Medicine, Seattle, Washington; UW Medicine Regional Burn Center, Seattle, Washington; University of Washington, Seattle, Washington; Department of Rehabilitation Medicine, University of Washington School of Medicine, Seattle, Washington; University of Washington, Seattle, Washington; UW Medicine Regional Burn Center, Seattle, Washington; University of Washington, Seattle, Washington; UW Medicine Regional Burn Center, Seattle, Washington; University of Washington School of Medicine, Seattle, Washington; UW Medicine Regional Burn Center, Seattle, Washington; University of Washington, Seattle, Washington; Department of Rehabilitation Medicine, University of Washington School of Medicine, Seattle, Washington; University of Washington, Seattle, Washington; UW Medicine Regional Burn Center, Seattle, Washington; University of Washington, Seattle, Washington; UW Medicine Regional Burn Center, Seattle, Washington; University of Washington School of Medicine, Seattle, Washington; UW Medicine Regional Burn Center, Seattle, Washington; University of Washington, Seattle, Washington; Department of Rehabilitation Medicine, University of Washington School of Medicine, Seattle, Washington; University of Washington, Seattle, Washington; UW Medicine Regional Burn Center, Seattle, Washington; University of Washington, Seattle, Washington

## Abstract

**Introduction:**

Patient-Reported Outcomes Measure System-10 (PROMIS-10) is a brief survey consisting of 10 items that evaluates patients’ global physical and mental health. PROMIS-10 was introduced into our burn clinic as a screening tool and serial measure of recovery. We aimed to assess the implementation of PROMIS-10 to improve its equitable use in outpatient clinics.

**Methods:**

The PROMIS-10 was implemented in 2014 as part of a packet of other screening tools. The staff were trained in its use. Patients completed English-language surveys via paper/pen upon arrival at each of their burn clinic appointments while waiting to be roomed. Survey results were reviewed by clinic providers and addressed as part of routine care. Survey answers were entered into REDCap and merged with demographic and burn injury information. Data were collected and analyzed using univariate and bivariable statistics to evaluate equitable implementation.

**Results:**

A total of 1,963 surveys were collected from 1,606 patients from January 2014 to December 2020 (Figure 1). After excluding patients with missing collection of survey and/or demographic data, our analysis included 1,072 patients. The mean number of surveys completed per visit was 0.53 (SEM=0.007) and 88% of patients completed 1 or more surveys. Patients who required intensive care during their index hospitalization or had an operation had higher mean surveys completed per visit (Highest Level of Care ICU=0.611 ± 0.02; Required an operation = 0.608 ± 0.015), whereas patients requiring an interpreter had lower mean surveys completed per visit (Required an Interpreter = 0.468 ± 0.031).

**Conclusions:**

Although the majority of patients completed the PROMIS-10 during at least one clinic visit, there is significant room for improvement given our intent to use it as a screening tool for every encounter and metric of recovery progress over time. Focus on equity in completion is needed (e.g., coordinating interpreter-assisted completion, availability of translated forms, person-assisted completion for limited English proficiency, medical record integration for telemedicine and progress tracking). We did note that patients with markers of high burn severity (ICU stay, requiring an operation) had higher rates of survey completion per visit. This is a patient population in which serial evaluation is particularly helpful in monitoring recovery.

**Applicability of Research to Practice:**

Use of PROMIS-10 can successfully be used as a screening and recovery progress metric in the outpatient setting. Equitable implementation should be assessed, and efforts to integrate such screening tools into the medical record should be considered.